# Effective prevention of pseudothrombocytopenia in feline blood samples with the prostaglandin I_2_ analogue Iloprost

**DOI:** 10.1186/s12917-015-0510-x

**Published:** 2015-08-06

**Authors:** Barbara Riond, Andrea Katharina Waßmuth, Sonja Hartnack, Regina Hofmann-Lehmann, Hans Lutz

**Affiliations:** Clinical Laboratory, Vetsuisse Faculty, University of Zurich, 8057 Zurich, Switzerland; Section of Epidemiology, Vetsuisse Faculty, University of Zurich, 8057 Zurich, Switzerland

**Keywords:** Feline EDTA blood, Iloprost, Platelets, Prostaglandin I_2_-analogue, Pseudothrombocytopenia, Platelet aggregates

## Abstract

**Background:**

In vitro platelet aggregation in feline blood samples is a well-known phenomenon in veterinary clinical laboratories resulting in high numbers of pseudothrombocytopenia. Several attempts have been made to prevent or dissolve platelet aggregates in feline blood samples and to increase the reliability of feline platelet counts. Prostaglandin I_2_ (PGI_2_) is the most powerful endogenous inhibitor of platelet aggregation but unstable. Iloprost is a stable PGI_2_ analogue. The aims of the present study were (1) to evaluate the anti-aggregatory effect of Iloprost on feline platelet counts and to determine a useful concentration to inhibit platelet aggregation in EDTA samples from clinically healthy cats, (2) to investigate the effect of Iloprost on hematological blood parameters, and (3) to determine stability of Iloprost in K3-EDTA tubes for up to 16 weeks. From 20 clinically healthy cats blood was drawn from the jugular vein and immediately distributed in a 1.3 ml K_3_-EDTA tube, and two 1.3 ml K_3_-EDTA tubes containing 20 ng and 200 ng Iloprost, respectively. A complete blood cell count was performed on the Sysmex XT-2000iV and the Mythic 18 on eight consecutive time points after collection. Blood smears were evaluated for the presence of PLT aggregates.

**Results:**

In the absence of Iloprost, pseudothrombocytopenia was observed in 50 % of the investigated samples that led to significantly decreased optical PLT counts by a mean of 105 x10^3^/μl, which could be prevented by the addition of 1 μL (20 ng) Iloprost leading to an increase in PLT counts by a mean of 108 x10^3^/μl.

**Conclusion:**

This is the first study showing an anti-aggregatory effect of the PGI_2_-analogue Iloprost in feline EDTA blood. In all clinically healthy cats investigated, pseudothrombocytopenia was prevented by adding Iloprost to EDTA tubes prior to blood collection. Furthermore, Iloprost was very useful in preventing falsely increased WBC counts in samples with platelet aggregates analyzed on impedance-based hematological instruments. Iloprost is preferable to PGI_2_ or PGE_1_ due to its stability and easy and safe handling properties. Cytological evaluations of blood smears as well as other hematological parameters were not influenced to a clinically significant degree by the presence of Iloprost.

## Background

In vitro platelet aggregation in feline blood samples is a well-known phenomenon resulting in pseudothrombocytopenia, with reported prevalences of 52 % [1], 62 % [2], and 71 % [3]. Several attempts have been made to prevent or dissolve platelet aggregates and decrease pseudothrombocytopenia in feline blood samples to increase the reliability of feline platelet counts. It has been shown that a citrate-based anticoagulant containing the platelet inhibitors theophylline, adenosine, and dipyridamole reduces feline platelet aggregation in blood samples more effectively than citrate or ethylenediaminetetraacetic acid (EDTA) [3]. Tvedten and Korcal investigated whether mixing blood with a vortex mixer could disaggregate platelet aggregates mechanically, but few of the investigated samples appeared to have all platelet aggregates dispersed [4]. Consequently vortex mixing does not seem to be a consistent means of correcting the problem of feline platelet aggregation. Prostaglandins are synthesized from arachidonic acid by cyclooxygenase in the cell membranes of nearly all tissues. Both prostaglandin E_1_ (PGE_1_) and prostaglandin I_2_ (PGI_2_) have platelet aggregation inhibitory activities, and PGE_1_ has been successfully used to inhibit platelet aggregates in platelet-rich feline plasma [5]. A combination of an optical platelet count together with adding PGE_1_ to EDTA blood collection tubes is recommended for platelet counting in feline patients with potential thrombocytopenia [6]. PGI_2_ is the most potent platelet aggregation inhibitor, but it is very unstable at physiological pH values and requires strict handling procedures [5]. Three decades ago, the stable PGI_2_ analogue Iloprost was synthesized. Iloprost is used as a therapeutic agent in human medicine to treat peripheral vascular diseases, severe pulmonary hypertension, and thrombangiitis obliterans [7]. Platelet aggregation and release reactions stimulated by aggregating agents such as arachidonic acid, collagen, or epinephrine are essentially abolished by nanomolar concentrations of Iloprost. The anti-aggregatory effect of Iloprost is based on an increase in cyclic adenosine monophosphate (cAMP) within the platelet. Platelets have high-affinity binding sites for PGI_2_ that can also bind Iloprost in a reversible, saturable manner. The formation of cAMP by the enzyme adenylate cyclase is mediated via these receptors. Increasing levels of cAMP within the platelet influence several processes involved in platelet activation, including the regulation of phospholipases and free cytosolic calcium levels [7]. Because of its chemical and biological stability, universal potent anti-aggregatory effect, and availability, Iloprost is more likely than PGI_2_ and PGE_1_ to be useful in preventing platelet aggregation in feline blood samples. The aims of the present study were (1) to evaluate the anti-aggregatory effect of Iloprost on feline platelet counts and to determine a useful concentration to inhibit platelet aggregation in EDTA samples from clinically healthy cats, (2) to investigate the effect of Iloprost on hematological blood parameters, and (3) to determine stability of Iloprost in K_3_-EDTA tubes.

## Methods

### Pilot study with different Iloprost concentrations

To test the overall effectiveness of Iloprost to prevent feline platelet aggregation, six different Iloprost amounts between 1 μg (50 μL of Ilomedin, Schering) and 20 ng (1 μL of Ilomedin) were tested in blood samples from five clinically healthy cats well known to build platelet aggregates in EDTA blood tubes (TVB 99/2007, 100/2007, and 101/2007 approved by the veterinary office of the canton Zurich). Just before blood collection, a 1 mL ampulla containing 20 μg of Iloprost (Ilomedin) was opened and a predetermined volume was pipetted into 1.3 mL K_3_-EDTA tubes (Sarstedt) with a micropipette (Calibra digital, Socorex Isba S.A.). Blood was collected with a needle (22 G, Neolus Terumo) and a 5 mL syringe (Omnifix, B. Braun) from the jugular vein. The needle was immediately removed after collection, and equal amounts of blood were placed in a K_3_-EDTA tube and three K_3_-EDTA tubes containing different Iloprost concentrations. The two persons involved in the blood collection process ensured that filling, closing, and mixing of all blood tubes occurred nearly simultaneously. All tubes were gently mixed for 2 min. A second blood collection was performed to test the remaining three Iloprost concentrations. For three of the five cats, blood collection was performed under deep sedation (10 mg/kg ketamine, Narketan, Vétoquinol AG in combination with 0.1 mg/kg midazolam, Dormicum, Roche Pharma AG).

The blood samples were analyzed at 15 min (min), 30 min, and 1, 2, 3, and 4 h (h) after blood collection. Between time points, the blood was mixed at room temperature on an automated mixer (Rock ‘n Roller 34201, Snijders Scientific B.V., AR). At each time point, blood samples were analyzed on the Sysmex XT-2000iV (Sysmex Corporation) for impedance and optical platelet counts and for total white blood cell (WBC) counts [8], and a blood smear was prepared and stained (HemaTek, Siemens).

Approval for collecting blood samples was given by the veterinary office of the canton Zurich, Switzerland (TVB number 99/2007, 100/2007, and 101/2007).

### Evaluation of the effect of 20 ng (1 μL) and 200 ng (10 μL) of Iloprost on 20 feline EDTA samples

Blood samples were next obtained from the jugular vein of 20 clinically healthy cats (TVB 99/2007, 100/2007, and 101/2007). Blood collection was performed as described for the pilot study. Immediately after collection, the needle was removed and equal amounts of blood were placed in 1.3 mL K_3_-EDTA tubes without Iloprost, with 1 μL (20 ng) Iloprost, and with 10 μL (200 ng) Iloprost, respectively. Blood collection was performed under deep sedation in 10 of the 20 cats. The blood samples were analyzed at 15 min and 1, 2, 3, 4, 6, 8, and 24 h after blood collection. Between time points, the blood was mixed on an automated mixer at room temperature (Rock ‘n Roller 34201, Snijders Scientific B.V). At each time point, the blood samples were analyzed on the Sysmex XT-2000iV (Sysmex Corporation) for a complete blood cell count including impedance and optical platelet counts, and a blood smear was prepared. Seven cats with platelet aggregates were additionally analyzed on the Mythic 18 instrument (Orphée SA) for total WBC counts at two time points [9]. The first measurement was immediately after blood collection, and the second was performed when platelet counts on the Sysmex XT-2000iV dropped under the lower reference limit (180 × 10^3^/μL) for the first time. All blood smears were assessed by one of the authors for the presence of platelet aggregates by applying the following scoring system per slide: 0, no aggregates; 1, aggregates of 2–5 platelets; 2, aggregates of 6–15 platelets; 3, aggregates of 16–50 platelets; and 4, aggregates of >50 platelets. Furthermore, blood smears were evaluated for estimation of WBC counts and to determine whether Iloprost has an effect of WBC and RBC morphology.

### Iloprost stability study

Iloprost stability was investigated over 16 weeks using blood samples from two clinically healthy cats known to develop platelet aggregates after each blood collection. Both cats had previously been used in the second experiment of the present study. Before study onset, 40 EDTA tubes (Sarstedt, 1.3 mL) were prepared with 1 μL (20 ng) of Ilomedin. Twenty tubes were stored at room temperature, and another 20 tubes were stored at 37 °C in an incubator. Blood collection was performed after 1, 2, 3, 6, 8, 12, and 16 weeks of storage. At each time point, 3 mL of blood was collected from the jugular vein as previously described and distributed equally into one K_3_-EDTA tube, one K_3_-EDTA tube with 1 μL (20 ng) Iloprost stored at room temperature, and one K_3_-EDTA tube with 1 μL (20 ng) Iloprost stored at 37 °C. The blood samples were analyzed 1, 2, 3, and 4 h after collection in the Sysmex XT-2000iV for optical platelet counts.

### Statistical analysis

Statistical analysis was performed with the software packages R and nlme [10]. Linear mixed effects models were used to model the outcome variable platelet counts, WBC, red blood cell (RBC), hematocrit (HCT), hemoglobin concentration (HGB), mean corpuscular hemoglobin (MCH), mean corpuscular volume (MCV), and mean corpuscular hemoglobin concentration (MCHC) measured on the Sysmex XT-2000iV, with the potential explanatory variables time, Iloprost administration (yes or no), aggregates (no: scoring index blood smear evaluation 0, 1; or yes: 2, 3, 4), an interaction term between Iloprost and aggregates as fixed effects, and cats as random effects to account for potential within-animal clustering. Model selection and validation were based on Akaike’s Information Criterion and the residuals for normality and homogeneity were checked visually. The results of the linear mixed effects models are given as effect sizes and their corresponding standard errors and *p*-values. To evaluate the changes in WBC counts analyzed with the Mythic 18 instrument (Orphée SA) at two time points, paired t-tests with and without Iloprost administration were performed.

Bar charts were made using GraphPad Prism (version 3.00 for Windows, GraphPad Software).

## Results

### Pilot study with different Iloprost concentrations

**Fig. 1:**
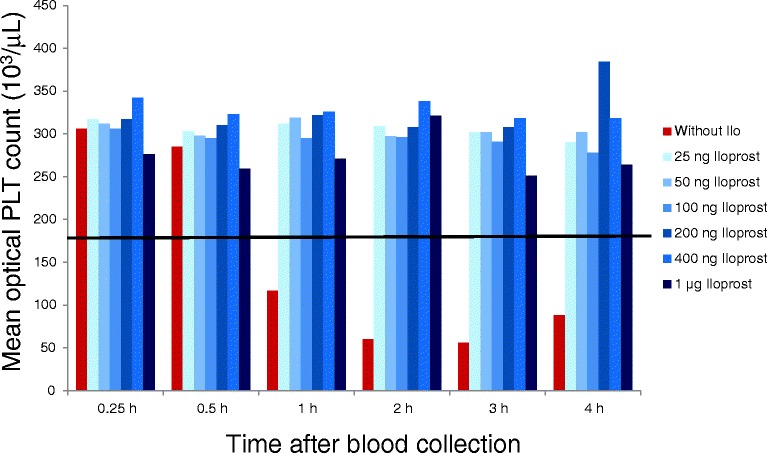
Bar chart showing results from the pilot study with different Iloprost concentrations. Mean optical platelet counts (10^3^/μl) from 5 cats with platelet aggregation without Iloprost and with 25 ng, 50 ng, 100 ng, 200 ng, 400 ng and 1 μg Iloprost at 0.25 h, 0.5 h, 1 h, 2 h, 3 h and 4 h after blood collection. The black line indicates the upper reference interval for feline platelets (180 x10^3^/ μL)

Within 4 h, mean platelet counts decreased in all EDTA tubes without Iloprost at timepoints 1 h, 2 h, 3 h and 4 h under the lower reference limit (180 × 10^3^/μL). In the EDTA tubes with Iloprost, mean platelet counts remained within the reference limits at each of the six tested Iloprost concentrations (Fig. 1). All tested Iloprost concentrations were effective to prevent pseudothrombocytopenia.

### Evaluation of the effect of 1 μL (20 ng) and 10 μL (200 ng) Iloprost on 20 feline EDTA samples

Fifty percent of the investigated feline EDTA samples without Iloprost showed pseudothrombocytopenia, with platelet counts under the lower reference limit at different time points. In EDTA tubes with Iloprost, pseudothrombocytopenia was prevented in all cases; however platelet aggregates were not completely dissolved. Blood smear evaluation of EDTA samples with Iloprost yielded platelet aggregation scores ranging from 0 to 2, whereas blood smears from EDTA samples without Iloprost had platelet aggregations scores ranging from 0 to 4. Iloprost had no effect on WBC and RBC morphology.

**Fig. 2:**
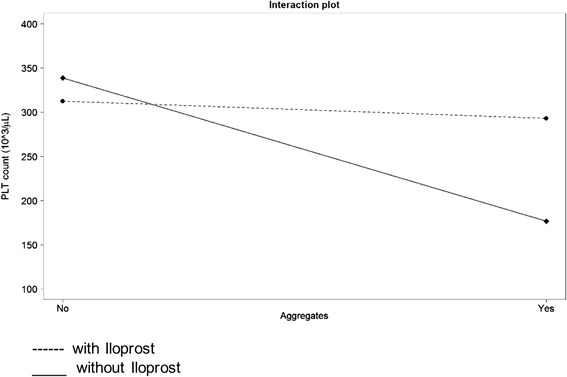
Interaction plot for optical platelet (platelet) counts in cats with (Yes) and without (No) platelet (platelet) aggregates. The black dashed line corresponds to Iloprost (Yes)

**Fig. 3:**
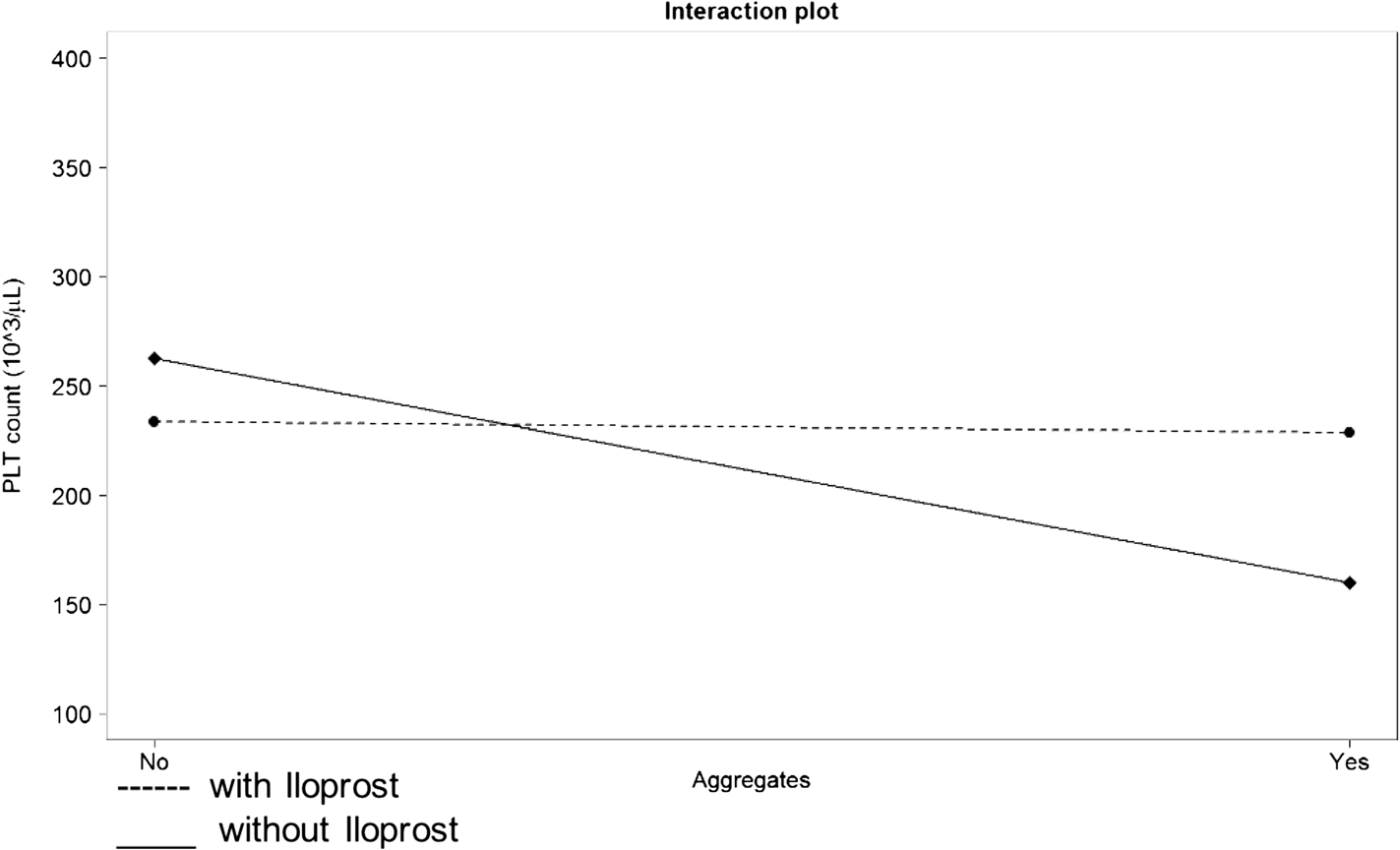
Interaction plot for impedance platelet (platelet) counts in cats with (Yes) and without (No) platelet (platelet) aggregates. The black dashed line corresponds to Iloprost (Yes)

The raw data of platelet counts measured by optical and impedance-based methods per time point, in the absence or presence of platelet aggregates and Iloprost administration are shown in Tables 1 and 2. The results of the linear mixed effects approach to assess potential effects of Iloprost, presence of aggregates, and an interaction between Iloprost and presence of aggregates as well as time on the optical and impedance-based platelet counts are shown in Table 3. In both methods, the optical and impedance-based platelet counts, the effect of Iloprost on increasing the platelet counts (by 108 × 10^3^/μL and 95 × 10^3^/μL) is mainly seen if aggregates are present, thus outweighing the effect of aggregates in decreasing platelet counts (−105 × 10^3^/μL and −94 × 10^3^/μL, respectively). This indicates an interaction effect between Iloprost administration and presence of aggregates, thus meaning that an effect of Iloprost on the PLT counts is mainly seen in the presence of aggregates but not in the absence. The interaction effect between Iloprost and presence of aggregates for each method is shown in Fig. 2 and Fig. 3. Over time, a slight decrease in platelet counts was seen. A significant difference between 1 μL (20 ng) and 10 μL (200 ng) Iloprost could not be found for either the impedance platelet count or the optical platelet count in a subsequent analysis using a linear mixed effects models to compare the effect of different amounts of Iloprost (Sysmex, *P* = 0.25; Mythic, *P* = 0.83).

**Table 1:** Means and standard deviations of optical platelet counts per time point, in the absence or presence of aggregates with and without Iloprost administration

Time in hours	Aggregates present									Aggregates absent								
	Ilo yes						Ilo no			Ilo yes						Ilo no		
	20 ng			200 ng						20 ng			200 ng					
	mean^1^	sd^1^	n	mean^1^	sd^1^	n	mean^1^	sd^1^	n	mean^1^	sd^1^	n	mean^1^	sd^1^	n	mean^1^	sd^1^	n
0.15	296	42	4	296	37	4	288	127	5	343	124	16	340	129	16	338	125	15
1	314	41	3	296	53	4	183	138	8	339	119	17	337	126	16	363	125	12
2	285	15	2	275	37	2	81	70	10	342	119	18	337	124	18	365	131	10
3	351	70	3	284	NA^2^	1	148	105	14	330	123	17	329	119	19	396	161	6
4	276	29	3	304	67	5	179	88	13	334	122	17	331	132	15	369	173	7
6	290	24	3	276	1	2	207	74	12	316	125	17	314	123	18	332	159	8
8	239	NA^2^	1	330	88	2	205	72	11	287	128	19	286	127	18	309	154	9
24	231	NA^2^	1	153	NA^2^	1	182	80	8	230	115	19	230	114	19	274	118	12

^1^ mean and standard deviation of platelet counts in 10^3^ x μL

^2^ 1 cat

**Table 2:** Means and standard deviations of impedance-based platelet counts per time point, in the absence or presence of aggregates with and without Iloprost administration

Time in hours	Aggregates present									Aggregates absent								
	Iloprost yes						Iloprost no			Iloprost yes						Iloprost no		
	20 ng			200 ng						20 ng			200 ng					
	mean^1^	sd^1^	n	mean^1^	sd^1^	n	mean^1^	sd^1^	n	mean^1^	sd^1^	n	mean^1^	sd^1^	n	mean^1^	sd^1^	n
0.15	266	85	4	278	68	4	276	148	5	310	104	16	300	120	16	295	95	15
1	281	85	3	257	83	4	171	134	8	295	112	17	295	109	16	294	99	12
2	142	81	2	192	22	2	66	57	10	271	111	18	258	113	18	288	113	10
3	282	136	3	154	NA^2^	1	136	100	14	234	100	17	246	120	19	280	80	6
4	176	45	3	249	119	5	168	83	13	238	118	17	236	111	15	252	112	7
6	175	21	3	186	1.4	2	187	84	12	215	113	17	215	115	18	237	108	8
8	150	NA^2^	1	303	165	2	181	90	11	191	97	19	188	90	18	237	81	9
24	140	NA^2^	1	83	NA^2^	1	152	79	8	137	61	19	141	70	19	202	78	12

^1^ mean and standard deviation of platelet counts in 10^3^ x μL

^2^ 1 cat

**Table 3:** Statistical analysis of optical platelet and impedance platelet counts

	Value (10^3^/μL)		SE (10^3^/μL)		*P*-value	
	Optical	Impedance	Optical	Impedance	Optical	Impedance
constant	332.3	287.9	25.5	21.0	<0.0001	<0.001
Ilo	−0.4	−25.8	6	7.1	0.9428	0.0003
Aggregates	−105.4	−94.4	8.1	9.6	<0.0001	<0.001
Time	−3.7	−4.9	0.3	0.3	<0.0001	<0.001
Interaction Ilo:Aggregates	108	95.3	11.3	13.5	<0.0001	<0.001

This table presents the results of a linear mixed effects model with PLT counts as outcome variable. The following variables were included as explanatory variables in the final model: administration of Iloprost (“Ilo”), presence or absence of aggregates (“Aggregates”), measurements taken over time (“Time”) as well as an interaction effect between Iloprost and presence of aggregates (“Interaction Test Ilo:Aggregates”). The results are given in the form of the mean effect size (“Value”) and their corresponding standard error (“SE”) as a measure of uncertainty and the p-values of each explanatory variable. The constant term or intercept indicates the value at which the fitted regression line crosses the y-axis or in other words corresponds to the mean PLT count if there would be no Iloprost administration, and no aggregates present at time point 0

No significant effect of Iloprost administration on WBC, RBC, HGB, HCT or MCV was found. Although significant (*p*-values of 0.0012 and 0.004), the effect of Iloprost administration on MCH and MCHC was considered to be clinically irrelevant with a decrease of - 0.052 pg (±0.016 pg) and - 0.12 g/dL (±0.04 g/dL). A significant increase over time for MCV, RBC, HCT, HGB and MCHC, albeit clinically not relevant, was observed.

In EDTA samples without Iloprost, mean WBC counts measured with the Mythic 18 increased strongly at the second measurement time point compared with the first. At the first time point mean WBC count was 8.87 × 10^3^/μL (±2.50 × 10^3^/μL), and at the second time point it was 15.49 × 10^3^/μL (±3.07 × 10^3^/μL 3.07). In contrast mean WBC counts in EDTA tubes with Iloprost remained completely stable at 8.66 × 10^3^/μL (±2.24 × 10^3^/μL, ± 2.21 × 10^3^/μL) at both time points. Based on paired t-tests, the difference between the two time points differed significantly in the samples without Iloprost (*p* < 0.0001) but not in the group with Iloprost administration. Blood smear evaluation from EDTA samples without Iloprost showed platelet aggregates in all cats at the second measurement time point; however no change in WBC count and distribution was observed compared to the blood smears from EDTA samples with Iloprost.

### Iloprost stability study

Results of the Iloprost stability experiment are displayed in Table 4. Optical platelet counts in EDTA tubes containing Iloprost at room temperature or in the incubator were higher than mean platelet counts in EDTA tubes without Iloprost. Mean platelet counts in EDTA tubes without Iloprost were below the lower reference limit at time point 2 h.

**Table 4:** Results of the stability study (measuring time point 2 h) for cat 1 and cat 2: Platelet counts in the EDTA tube, the EDTA tube + 20 ng Iloprost stored at room temperature and the EDTA tube + 20 ng Iloprost stored at 37 °C

Optical platelet counts (10^3^/μL)	EDTA		EDTA + 20 ng Iloprost stored at room temperature		EDTA + 20 ng Iloprost stored at 37 °C	
	cat 1	cat 2	cat 1	cat 2	cat 1	cat 2
Week 1	29	91	292	480	296	461
Week 2	25	22	324	463	280	456
Week 3	11	77	323	528	323	535
Week 6	17	30	358	476	394	477
Week 8	16	21	296	462	313	478
Week 12	33	20	339	448	372	461
Week 16	16	20	288	370	299	395

## Discussion

This is the first study showing a strong anti-aggregatory effect for the synthetic PGI_2_ analogue Iloprost on feline platelet counts. In clinically healthy cats, pseudothrombocytopenia was completely prevented in all blood samples in EDTA tubes containing Iloprost. Without Iloprost, if significant numbers of platelet aggregates were present (score of > 1) in an EDTA blood sample, the mean optical platelet count decreased by 105 × 10^3^/μL. This decrease was completely counterbalanced by the presence of Iloprost in the EDTA tube. Iloprost did not affect optical platelet counts in samples without aggregates or with score 1 aggregates. For impedance platelet counts, the effect of Iloprost on aggregates was similar.

Iloprost effectively prevented pseudothrombocytopenia in the present study, but it did not dissolve platelet aggregates completely. Cats with optical platelet counts that remained within the reference intervals during the evaluation period nevertheless showed occasionally small platelet aggregates in blood smears, which were classified according to the scoring index as 1 or 2. In blood smears from EDTA samples with Iloprost, these platelets were observed to retain their granules over the evaluation period, and appeared morphologically round. In contrast, platelets that formed aggregates in samples without Iloprost lost their shape, formed pseudopods, and lost their granules. The scoring index used in this study to judge the presence of platelet aggregates in the blood smear was limited in that only the size, but not the frequency of aggregates, was investigated. If a single platelet aggregate with a score of 2 (6–15 platelets) was identified, the sample was categorized as having a platelet aggregate score of 2. In feline samples with unaffected optical platelet counts, platelet aggregates consisted mainly of only six to seven platelets. The blood smear evaluation technique used in this study seemed to be a very sensitive method for detecting platelet aggregates, particularly if the feathered edges of the smear were examined. From this observation it can be speculated that a few platelet aggregates consisting only of a small number of platelets do not influence the optical platelet count of the Sysmex XT-2000iV.

Similar to PGI_2_, PGE_1_ has platelet anti-aggregatory properties [5] previously showed that feline platelets exposed to PGE_1_ become immediately and persistently nonreactive to agonists, which prevents platelet aggregation and consequently improves platelet count accuracy. Tvedten and Johansson [6] demonstrated higher feline platelet counts in feline samples containing PGE_1,_ although a complete inhibition of aggregation could not be achieved. On blood smear evaluation, PGE_1_-treated samples had occasional platelet aggregates, but many more and larger aggregates were seen in EDTA samples. Although PGE_1_ has been shown to successfully prevent in vitro platelet aggregation in blood samples from cats, it is not used in daily laboratory hematology testing because of its poor stability.

In vivo, PGI_2_ is one of the main platelet anti-aggregatory agents. Similar to PGE_1_, PGI_2_ is characterized by poor stability; it has a half-life of approximately 2 min at 37 °C and pH of 7.4, and its activity is gone in 20 min at 22 °C [11]. Use of PGI_2_ in laboratory hematology testing is not practical. The synthetic PGI_2_ analogue Iloprost is both qualitatively and quantitatively more effective as an inhibitor of platelet aggregation than either PGI_2_ or PGE_1_ [7]. Furthermore, Iloprost is available as Ilomedin, an intravenous drug in human medicine. Ilomedin is readily usable due to its pharmacological formulation. In the present study, all Iloprost concentrations tested showed the same strong anti-aggregatory effect on feline platelets. Concentrations for PGI_2_ causing 50 % inhibition of ADP-induced platelet aggregation have been reported for different species other than the cat, and range from 0.5 to 3.7 ng/mL [11]. Due to the specific peculiarities of the feline platelet, which appears to be more readily activated and aggregated compared to other species [12], the authors assumed that higher amounts of Iloprost would be needed to inhibit aggregation of feline platelets. To investigate the effect of Iloprost in a reasonable number of cat blood samples, 1 μL (20 ng) and 10 μL (200 ng) of Iloprost were chosen. Both amounts achieved the same effect, and no significant difference between EDTA tubes with 1 μL vs. 10 μL was observed. For this reason, the Iloprost stability study and Iloprost testing in clinical cases were performed with 1 μL (20 ng) Iloprost. The smaller amounts are likely sufficient to prevent pseudothrombocytopenia, but amounts greater than 1 μL (20 ng) and 10 μL (200 ng) would be needed to completely prevent platelet aggregation.

The use of Iloprost in veterinary hematology would be beneficial for preventing pseudothrombocytopenia in feline EDTA samples. Adding Iloprost to EDTA tubes used for hematological analysis of feline blood samples should improve the reliability of platelet counts. The main weaknesses of PGI_2_ and PGE_1_ for routine application are their poor stability and complex handling procedures. In contrast, Iloprost can be stored at room temperature, and is easy to handle and immediately useable. In the present study Iloprost was stored for up to 16 weeks in EDTA tubes at room temperature and at 37 °C. The effectiveness of Iloprost on platelet aggregates persisted over the entire storage time. Iloprost did not significantly affect hematological parameters, cytological evaluation of the blood smears and the observed decreases in MCH and MCHC were negligible.

It is a well-known phenomenon in feline blood samples that platelet aggregates can falsely increase WBC counts and decrease platelet counts in impedance-based hematological instruments [13, 14]. In the present study, false-positive leucocytosis was observed in six out of seven EDTA samples, whereas false leucocytosis did not occur in the EDTA samples with Iloprost. This observation is important because impedance-based instruments are widely used in veterinary practices, and false WBC counts due to platelet aggregation are a common finding. Using EDTA tubes with Iloprost for hematological analysis on impedance instruments would improve the reliability of both WBC counts and platelet counts in cats.

## Conclusions

In conclusion, this is the first study showing an anti-aggregatory effect of the PGI_2_-analogue Iloprost in feline EDTA blood. In all clinically healthy cats investigated, pseudothrombocytopenia was prevented by adding Iloprost to EDTA tubes prior to blood collection. Furthermore, Iloprost was very useful in preventing falsely increased WBC counts in samples with platelet aggregates analyzed on impedance-based hematological instruments. Iloprost is preferable to PGI_2_ or PGE_1_ due to its stability and easy and safe handling properties. Cytological evaluations of blood smears as well as other hematological parameters were not influenced to a clinically significant degree by the presence of Iloprost.

## Abbreviations

PLT: Platelets
WBC: White blood cells
EDTA: Ethylenediaminetetraacetic acid
h: Hours
min: Minutes
PGE1: Prostaglandin E1
PGI2: Prostaglandin I2
CAMP: Cyclic adenosine monophosphate
HCT: Haematocrit
HGB: Hemoglobin concentration
MCH: Mean corpuscular hemoglobin
MCV: Mean corpuscular volume
MCHC: Mean corpuscular hemoglobin concentration
